# Comment on Tsai, Y.-C., et al. Association of Stress-Induced Hyperglycemia and Diabetic Hyperglycemia with Mortality in Patients with Traumatic Brain Injury: Analysis of a Propensity Score-Matched Population. *Int. J. Environ. Res. Public Health* 2020, *17*, 4266

**DOI:** 10.3390/ijerph18052533

**Published:** 2021-03-04

**Authors:** Eduardo Mekitarian Filho

**Affiliations:** Santa Catarina Hospital, Av. Paulista, 200, Sao Paulo 01310-000, Brazil; emf2002@uol.com.br; Tel.: +55-11-30164317

I read carefully the great study published by Tsai, Y.-C. et al. [1], published in the June issue of International Journal of Environmental Research and Public Health.

Following traumatic brain injury (TBI), hyperglycemia may contribute to lactic acidosis in brain tissue, resulting in neuronal injury mainly by free-scavengers production. Hyperglycemia is associated with higher morbidity and mortality in patients, in both pediatric and adult settings. Hyperglycemia may be interpreted with diabetic hyperglycemia (DH) or stress-induced hyperglycemia (SIH) [2]. DH and SIH are diagnosed when serum glucose concentrations are ≥ 200 mg/dL in patients with and without diabetes mellitus (DM), respectively. A combination of inflammatory cytokines with microvascular diabetic lesions may be responsible for this alteration [3].

Since Chiaretti’s first study, published in 1998 [4], the association between SIH and bad outcomes in TBI has been known. The authors, in the paper from Tsay [1], found strong data evidencing SIH with mortality (odds ratio 1.91; 95% confidence interval of 1.12–1.23). Patients with diabetes are commonly excluded, but not in this study, and their increase in morbidity and mortality were not statistically significant.

The concern of neurologic sequelae was reported by Huang et al. [5] in a paper that evaluated mortality depending on many factors and hyperglycemia. Patients with 10 days or more of mechanical ventilation had greater body mass index (BMI) and were hyperglycemic. Indeed, this raises the chance of *delirium* in the intensive care unit (ICU). In a paper from Bedry et al. [6], hyperglycemia at admission also predicted bad brain injury.

Tsai, Y.-C. et al. [1] brought light to a very important issue that deserves to be studied, in children and/or adults, and it seems that hyperglycemia plays the role of a villain in TBI recovery.
